# Applications of artificial intelligence for machine- and patient-specific quality assurance in radiation therapy: current status and future directions

**DOI:** 10.1093/jrr/rrae033

**Published:** 2024-05-27

**Authors:** Tomohiro Ono, Hiraku Iramina, Hideaki Hirashima, Takanori Adachi, Mitsuhiro Nakamura, Takashi Mizowaki

**Affiliations:** Department of Radiation Oncology, Shiga General Hospital, 5-4-30 Moriyama, Moriyama-shi 524-8524, Shiga, Japan; Department of Radiation Oncology and Image-Applied Therapy, Graduate School of Medicine, Kyoto University, 54 Kawahara-cho, Shogoin, Sakyo-ku, Kyoto 606-8507, Japan; Department of Radiation Oncology and Image-Applied Therapy, Graduate School of Medicine, Kyoto University, 54 Kawahara-cho, Shogoin, Sakyo-ku, Kyoto 606-8507, Japan; Department of Radiation Oncology and Image-Applied Therapy, Graduate School of Medicine, Kyoto University, 54 Kawahara-cho, Shogoin, Sakyo-ku, Kyoto 606-8507, Japan; Department of Radiation Oncology and Image-Applied Therapy, Graduate School of Medicine, Kyoto University, 54 Kawahara-cho, Shogoin, Sakyo-ku, Kyoto 606-8507, Japan; Division of Medical Physics, Department of Information Technology and Medical Engineering, Human Health Sciences, Graduate School of Medicine, Kyoto University, 53 Kawahara-cho, Shogoin, Sakyo-ku, Kyoto 606-8507, Japan; Department of Radiation Oncology and Image-Applied Therapy, Graduate School of Medicine, Kyoto University, 54 Kawahara-cho, Shogoin, Sakyo-ku, Kyoto 606-8507, Japan

**Keywords:** artificial intelligence, machine learning, machine-specific quality assurance, patient-specific quality assurance

## Abstract

Machine- and patient-specific quality assurance (QA) is essential to ensure the safety and accuracy of radiotherapy. QA methods have become complex, especially in high-precision radiotherapy such as intensity-modulated radiation therapy (IMRT) and volumetric modulated arc therapy (VMAT), and various recommendations have been reported by AAPM Task Groups. With the widespread use of IMRT and VMAT, there is an emerging demand for increased operational efficiency. Artificial intelligence (AI) technology is quickly growing in various fields owing to advancements in computers and technology. In the radiotherapy treatment process, AI has led to the development of various techniques for automated segmentation and planning, thereby significantly enhancing treatment efficiency. Many new applications using AI have been reported for machine- and patient-specific QA, such as predicting machine beam data or gamma passing rates for IMRT or VMAT plans. Additionally, these applied technologies are being developed for multicenter studies. In the current review article, AI application techniques in machine- and patient-specific QA have been organized and future directions are discussed. This review presents the learning process and the latest knowledge on machine- and patient-specific QA. Moreover, it contributes to the understanding of the current status and discusses the future directions of machine- and patient-specific QA.

## INTRODUCTION

Recently, artificial intelligence (AI), including machine learning (ML), has been applied in various fields. The concept of AI was conceived in 1956, and several applications of computer-aided diagnosis were reported in the field of radiology after the first and second AI booms [1]. The current AI boom in 2024, often regarded as the third wave, has experienced explosive growth in popularity as compared with the previous periods, especially with the widespread adoption of deep learning (DL). Improvements in computer performance and development environments are believed to have encouraged the adoption of this technology. Developments in AI are expected to enhance the efficiency of radiotherapy through automation and enable the detection of phenomena that are challenging to predict using conventional methods. Recently, Philippe *et al*. [2] surveyed the applications of DL for radiotherapy and revealed that a rise in the number of papers related to the same has been published since 2012. In the radiotherapy workflow, they mentioned the potential applications of DL, including patient consultation, planning image acquisition, target and structure segmentation, treatment planning, treatment delivery and follow-up. AI has led to the development of various techniques for automated segmentation and planning, thereby significantly enhancing treatment efficiency [3–8]. Kawamura *et al.* [9] provided an overview of AI in radiotherapy from the perspective of a radiation oncologist. They expect to reduce the treatment planning time through AI auto-segmentation or other techniques, thereby increasing the time available for patient care.

Although the effectiveness of AI in the overall radiotherapy workflow has been reviewed, few review articles have specifically focused on the individual steps in the workflow. Quality assurance (QA) is one such crucial step for proper radiotherapy delivery and can be categorized as machine- and patient-specific QA. For machine-specific QA, the AAPM task groups (TG) 142 and 198 recommended QA procedures for medical accelerators [10, 11], while TG 106 recommended accelerator beam data commissioning equipment and procedures [12]. Based on these guidelines and recommendations, the safety of radiotherapy equipment must be guaranteed, many of which necessitate substantial effort and adequate experience. Traditionally, the quality of patient-specific QA for intensity-modulated radiation therapy (IMRT) or volumetric-modulated arc therapy (VMAT) has been determined using measurement-based QA methods [13]. TG 219 has published calculation-based QA methods [14]. To ensure that patient plans are conducted appropriately, it is necessary to address the mechanical errors during actual irradiation and develop efficient methods for evaluating the results of patient-specific QA, considering the wide variety of methods currently available. Several articles on the applications of AI for machine- and patient-specific QA, including those for predicting beam data to assess the safety of treatment equipment [15–21], as well as methods for predicting multi-leaf collimator (MLC) position errors during actual irradiation [22–29] and gamma passing rate (GPR) of IMRT or VMAT plans [30–40]. Review articles on QA for radiotherapy have been published but are scarce [6–8]. However, the number of such applications has increased rapidly since the third AI boom, leading to a variety of novel methods being currently in use. Furthermore, novel optimization techniques for treatment planning and quality management methodologies, all grounded in AI technologies, have been reported [41–43]. In order to embrace the rapid evolution of AI technology that has been occurring within a few mere years, there is a need to understand the strategies and directions of machine- and patient-specific QA by reviewing currently available studies.

This literature review aims to organize the current status of AI applications in machine- and patient-specific QA and discuss its future directions. In the first section, the input data or ML models, including that of non-DL and DL, for the application of AI in the field of radiotherapy are presented with a focus on practical applications. Subsequently, various reports on machine- and patient-specific QA were reviewed. Finally, the future directions based on the reviewed applications are discussed, addressing the latest AI technologies. Through this review article, it is hoped that the current status of QA in radiotherapy, while taking into consideration the rapidly expanding AI technology scene, will be summarized and its clinical applicability at clinical institutions be clarified.

## SEARCHING STRATEGY

This literature review utilizes PubMed to survey primarily articles on machine- and patient-specific QA using AI between January 2019 and December 2023. This study primarily surveys studies from the last 5 years to focus on the latest knowledge, given the rapid development of AI technology. However, a few key literature surveys also include studies published before 2019 as well. The main keywords used in the search were ‘radiotherapy,’ ‘radiation therapy,’ ‘artificial intelligence,’ ‘machine learning,’ ‘deep learning’ and ‘quality assurance.’ Articles related to machine- or patient-specific QA were manually selected from the retrieved literature.

## ARTIFICIAL INTELLIGENCE ALGORITHMS

**Fig. 1:**
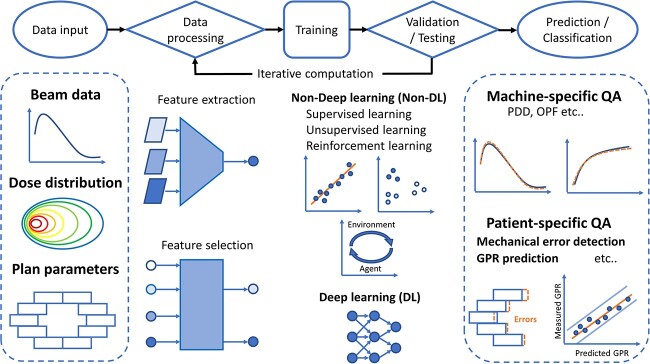
Learning process of application of AI for QA. Data input, beam data, dose distribution, and plan parameters are specific for machine- and patient-specific QA in the radiotherapy field. Abbreviations: QA, quality assurance; PDD, percent depth dose; OPF, output factor; MLC, multileaf collimator; GPR, gamma passing rate.

Although AI is a closely related field, it has distinct meanings and applications [44]. AI refers to a broad field of computer science that aims to create intelligent machines capable of emulating human-like intelligence. ML is a subset or subfield of AI that focuses on the development of algorithms and statistical models. ML algorithms enable machines to learn from data and make predictions or decisions without explicit programming. When using ML, the input data, learning methods, and validation are crucial. These elements directly impact the performance and reliability of a model. In addition, DL exists as a subset of ML. In this review article, conventional ML is defined as non-DL. Figure 1 shows the learning process of the application of AI to machine- and patient-specific QA in radiotherapy. The machine beam data, dose distribution, electronic portal imaging device (EPID) images, and plan parameters were used as input data. Prior to model training, data processing, including feature extraction and selection, was performed to prepare and clean the raw input data. For model training, validation, and testing, several types of ML methods, including that of non-DL and DL, were used according to their purpose. Iterative computations are performed to obtain the best model. The details of this process are as follows.

### Feature extraction and feature selection

Input data can significantly improve the performance and accuracy of the output prediction and classification [45, 46]. Generally, features are required to effectively explore for analysis purposes. Input data were processed using feature extraction and selection [47]. In contrast to feature selection, feature extraction typically requires the transformation of input data into features that possess robust pattern recognition capabilities. For simple feature extraction, values and indicators that have been shown to have a relationship with the outputs or are considered useful as predictors or classifiers are often used. For example, beam data from the Linear accelerator (Linac), plan complexity parameters, and plan dose parameters were used as input features. Osman *et al.* [24] predicted MLC positional accuracy from machine-recorded log files using a DL model of an artificial neural network (ANN). The predefined input data included fourteen parameters, including leaf position, dose fraction, segment number, beam of flag/state, gantry angle, carriage position, leaf gap, leaf speed and leaf status. These parameters were extracted from the planning data in the log files. However, when the input data has high-dimensional information, such as those from computed tomography (CT) and magnetic resonance (MR) images or dose distribution, features should be extracted to realize a low-dimensional vector using radiomics or dosiomics approaches [45, 48, 49]. Hirashima *et al*. [34] used dosiomic features to predict the GPR of VMAT plans using a non-DL model of eXtreme Gradient Boosting (XGBoost). They extracted 851 dosiomic features of the patient geometry using the PyRadiomics platform, with the extracted features including shape, statistical information, and texture.

The goal of feature selection is to improve the performance and robustness of the model, reduce redundant data, and potentially enhance the interpretability of the model. As applications in radiotherapy decision-making, non-DL models of Bayesian network models have been used to detect errors in radiotherapy plans [50, 51]. Feature selection methods, including non-DL models of principal component analysis (PCA), random forest, gradient boosting, and support vector machines (SVM), can be divided into feature ranking and subset selection models, based on the type of output. For feature ranking, such a non-DL model considers the correlation with the output by varying the weight of each factor. Valdes *et al*. [30] predicted the GPR of IMRT plans from 78 predefined input data points, including the plan complexity parameter or fractional monitor unit (MU), using non-DL models of Poisson regression with the Lasso regularization model. The 78 predefined input data points were selected using PCA [52]. Using PCA, the dimensions in which the plan complexity parameters had the largest variance were found in a visualized two-dimensional map. Finally, Valdes *et al.* used PCA only for the visualization of plan complexity parameters and Lasso regularization for performing feature selection.

For a large number of features, feature reduction is pivotal in reducing the amount of input data using subset selection models [47], especially when the amount of input data is large. Feature selection is crucial in reducing the amount of input data when developing a predictive model. The goal of feature selection is to improve the performance and robustness of the model, reduce redundant data, and potentially enhance the interpretability of the model. Ma *et al*. [19] classified the delivery errors of IMRT plans using structural similarity index measure (SSIM) subindex maps from radiomic features on portal dosimetry. They simulated machine-related errors and obtained 276 radiomic features. Subsequently, 20 ranked features were selected using a non-DL model of the SVM recursive feature elimination method [53]. Wall *et al.* [35]. selected features, including 241 plan complexities and plan parameters, to predict the GPR for 500 VMAT plans. They quantified and ranked the relative importance of each feature using non-DL models of forests of extremely randomized decision trees (Extra Trees), mutual information, and linear regression. They observed that the most important feature was the same for each selection model and that there were common features in the top five ranked features. For a large number of features, feature reduction is key in reducing the amount of input data using subset selection models [47]. Using a small number of features, Anetai *et al.* [54] confirmed that smaller information sufficiently provides clustering of dose distribution using a coefficient tensor of spherical harmonics using the Isomap method based on manifold learning. Thus, selecting a subset of the most crucial features has the potential to streamline the interpretation and visualization of data, reduce training and utilization times, and enhance the overall performance and robustness of the predictive model [55]. The review also showed that conventional ML methods, which are non-DL, are widely used for feature extraction and selection.

### Machine learning methods

Essentially, ML algorithms empower systems to improve their performance by using a computer to learn some features from data. Here, ML is divided into two categories of non-DL, including supervised learning, unsupervised learning, and reinforcement learning, and DL. The third wave of the AI boom has led to a proliferation of reports concerning DL within the realm of ML. However, it is important to note that there is also a substantial volume of reports focusing on non-DL methodologies. Hence, the DL method is not the only optimal approach for achieving performance in a specific context within radiotherapy. As evidenced by Li *et al*. [56], a basic linear regression surpassed the performance of recurrent neural networks for respiratory tracking.

### Non-deep learning methods

With regard to non-DL methods, the present study mainly focused on supervised, unsupervised, and reinforcement leaning. Supervised learning is a type of ML in which an algorithm learns from labeled training data to make predictions or decisions without explicit programming. Logistic regression, random forest, SVMs, and k-nearest neighbors (kNNs) have been used as statistical learning algorithms [57]. For example, in the field of radiotherapy, non-DL models on supervised learning have been used to predict clinical outcomes from biochemical markers or estimate tumor position from multiple anatomical features [58, 59]. In machine- and patient-specific QA in radiotherapy, these algorithms are often used to predict or classify mechanical errors or GPR as labeled training data. Zhao *et al.* [17] performed beam data modeling using supervised learning. They predicted the percentage depth doses (PDDs) and profiles of different field sizes using 43 beam datasets. They employed the Scikit-learn ML toolkit, including SVMs and kNNs, for model training [60]. Sun *et al*. [61] predicted the MU for a proton therapy treatment machine using three supervised learning methods, namely, Random Forest, XGBoost and Cubist. Here, XGBoost is a class of gradient-boosted regression trees [62] and Cubist is a tree-based model similar to Random Forest [63]. To predict MU, the output factor (OPF) of 23 clinical fields was set as the input, which was then trained and validated using 1754 fields. Recently, a light gradient boosting machine (LightGBM) was used to predict GPR based on radiomic features [64]. LightGBM is a data model based on a gradient-boosted regression tree and is designed to efficiently handle large datasets and high-dimensional feature spaces [65]. Therefore, it is expected to be used as an efficient ML model.

Unsupervised learning is a type of ML in which an algorithm is trained on a dataset without labeled output data. Specifically, ‘supervised’ or ‘unsupervised’ indicates whether the labels of identification automatically/manually correspond to the data. In radiotherapy, unsupervised learning is primarily used for clustering and anomaly detection. Azmandian *et al.* [66] developed an error detection method using a k-means clustering algorithm based on historical prostate plans. They used 1000 plans to build clusters, and 650 plans to test the proposed method. They identified eight clusters that accurately described the data.

Another non-DL method, reinforcement learning is a type of ML paradigm in which an agent learns to make sequences of decisions by interacting with the environment to achieve a specific goal. Although reinforcement learning has been applied in radiotherapy plan optimization [67] and beam orientation decisions [68], it is not widely used in ML in the radiotherapy field, and future developments are expected [69].

### Deep learning method

DL is a subfield of ML that focuses on ANNs and is inspired by the structure and function of the human brain. Compared with non-DL models, DL has a multilayer structure, and features can be automatically extracted from low- and high-dimensional data. Furthermore, it is possible to implement a learning process using a single DL model. DL can be used for either supervised, unsupervised, or reinforcement learning depending on the purpose. They have gained significant attention and popularity in recent years because of their remarkable ability to learn and represent complex patterns and features in data [2, 70]. As supervised learning, Kimura *et al*. [28] developed a mechanical error detection system for 161 prostate VMAT beams. For the error simulation, they used dose distributions containing nine types of errors, including MLC positioning, gantry rotation, radiation output, and phantom setup errors. To implement unsupervised learning, they adopted a variational autoencoder (VAE) [71]. The areas under the curves (AUC) of the VAE-based method, supervised convolutional neural network (CNN), and gamma analysis were found to be 0.699, 0.921 and 0.669, respectively. They concluded that the VAE-based method had the potential to detect errors in VMAT plans. Regarding reinforcement learning, Hrinivich *et al.* [72] developed a VMAT machine parameter optimization approach based on deep Q-reinforcement learning using 40 retrospective prostate cancer plans. A convolutional deep-Q network used clinical patient contours and optimized VMAT plans with a period of 1.5 ± 0.2 sec per slice. They concluded that the developed approach rapidly optimized the VMAT plans and generated sequences of deliverable machine parameters without adjusting the optimization objectives. Thus, it is widely used in machine- and patient-specific QA for radiotherapy. Bedford *et al.* [27] detected delivery errors during real-time portal dosimetry using a recurrent deep neural network. They investigated the VMAT of six patients with prostate cancer and introduced four different errors, namely, MU, aperture opening, aperture shift, and air gap. They found the neural network reduced the rate of false negative results from 0.36 to 0.24 when compared with conventional threshold techniques. Tomori *et al*. [32] predicted the GPR for 60 patients with prostate cancer with IMRT plans using deep 15-layer CNNs. They used input data, including the volumes of the PTV, rectum and overlapping region, measured area, and MU values for each field. They found that the GPR prediction accuracies at 2%/2, 3%/2 and 3%/3 mm were 1.9%, 1.3% and 1.1%, respectively. DL has the potential to generate more optimal models than non-DL models and is expected to be used further in the future.

### Data training, validation and testing

Data training, validation, and testing are essential processes in utilizing AI [73]. During the data-training phase, the ML model learns from a labeled dataset and provides an optimized model. The model generation procedure depends on the learning method. It is known that the number of datasets affects the accuracy of the model. In the validation phase, the appropriateness of the generated models was evaluated, including whether they caused an overestimation. Through this validation phase, the hyperparameters of model generation were properly tuned. The validation methods include hold-out, cross-validation, leave-one-out and stratified k-fold cross-validation. During the testing phase, the performance of the generated model was independently verified. In general, training and validation data should be separated from the test data for rigorous model training and testing. Cho *et al*. [74] determined the optimum size of the training dataset necessary to achieve high classification accuracy for CT images into six anatomical classes using a CNN. They trained six different sizes of training datasets, namely 5, 10, 20, 50, 100 and 200, and tested their classification accuracy. They observed that the largest training dataset (200) achieved the highest classification accuracy. Nemoto *et al*. [75] observed that a sample size of over 200 cases showed a stable Dice similarity coefficient for automatic segmentation of various organs using the U-Net algorithm. Although a large number of datasets are ideal, data accumulation may be difficult depending on the aspect of the study. When the number of data points is small, it is important to use appropriate validation methods and sort the datasets. Potter *et al*. [76] detected and classified errors in 13 IMRT plans (88 fields in total) using an ANN. Although they did not have sufficient data to evaluate the test dataset, they assessed the generalizability of the models through cross-validation.

## APPLICATION FOR MACHINE-SPECIFIC QUALITY ASSURANCE

Several procedures are recommended by AAPM TG142 and TG198 [10, 11]. Table 1 summarizes the studies applying AI for machine-specific QA. The learning model employed non-DL and DL. Li *et al.* [15] developed a time-series prediction model for machine-specific daily QA. They used ANNs and autoregressive moving averages (ARMA) to predict daily QA, including output, beam flatness, symmetry, and transverse symmetry, for 5-years of daily Linac QA data. They found that the prediction errors of the mean square errors were ~0.1 with ANN and concluded that the ANN time-series predictive model had more advantages over ARMA in terms of accuracy and effectiveness. Naqa *et al*. [16] developed an automated machine-specific QA method using support vector data description (SVDD) and SVM. They categorized some clusters that identified the tolerance of AAPM TG-142 for gantry sag, radiation field shift, and MLC offset with a total of 119 EPID images from eight Linacs and seven institutions. They found that the outliers of the clusters were 2.5%, 2.5% and 0.34% for gantry sag, radiation field shift, and MLC offset, respectively. They concluded that ML-based SVDD clustering is promising for developing reliable and reproducible automated QA tools. Douglass *et al*. [18] predicted the Winston Lutz (WL) images on an EPID using U-net. U-net was used to train the model on 1500 synthetic WL images. Using this model, ball bearing and MLC field segmentations were performed with mean Dice coefficients of 0.964 and 0.994, respectively. They found that the prediction of the ball-bearing locations was shown to correlate better with manual annotations than with Canny edge detection algorithms. They concluded that the WL image prediction approach had the potential to be applied to the automatic analysis of routine machine QA. Zhao *et al.* [20] predicted OPFs, including small fields, from 13 different MLC field sizes of 5–100 mm^2^. They used five different algorithms, including kernel ridge regression, random forest regression, decision tree regression with AdaBoost, gradient boosting regression and voting regression, and found that the predicted OPF had a mean relative error of 0.38 ± 0.39% (maximum: 3.62%). They observed that tree-based models yielded superior results compared with other ML methods. They concluded that the OPF prediction may serve as an input for dose calculations to overcome the limitations of small-field uncertainties. Liu *et al*. [21] predicted beam data, including PDDs and off-center ratios (OCRs), from golden beam data and 14 clinical beam datasets. They used neural representation learning and observed that the average mean absolute errors were <0.6% for PDDs and OCRs. Predicting the beam data is expected to contribute to reducing measurement times. The application of AI to machine QA is expected to improve the efficiency of beam data measurements and predict uncertain values based on actual measurements.

**Table 1:** Summary of the studies on applying AI for machine-specific QA

Reference	Year	Procedure	Learning models		Data set and input	Main result
			Non-DL	DL		
Li *et al.* [15]	2017	Output, beam flatness, symmetry, and transverse symmetry	ARMA	ANN	Daily QA data over 5 years	Prediction errors of MSE were around 0.1 with ANN
El Naqa *et al.* [16]	2019	Gantry sag, radiation field shift, MLC offset	SVM and SVDD	-	199 data on EPID images	Outliers of clusters were 2.5%, 2.5%, and 0.34% for gantry sag, radiation field shift, and MLC offset, respectively
Zhao *et al.* [17]	2020	PDDs	SVM and kNN	-	43 beam data sets	Predicted PDD had a mean absolute error between 0.19 and 0.35%
Douglass *et al.* [18]	2021	Winston Lutz analysis	-	U-net	1500 images	Winston Lutz's image was segmented with a mean dice coefficient of 0.964 and 0.994 for ball bearing and MLC field, respectively
Zhao *et al.* [20]	2022	OPF	Kernel ridge, random forest, Decision tree, Gradient boosting, Voting regressions	-	13 different MLC field sizes	Predicted OPF was a mean relative error of 0.38 ± 0.39% (maximum: 3.62%)
Liu *et al.* [21]	2023	PDDs and OCRs	-	Neural representation learning	Golden beam data and 14 clinical beam data	Averaged mean absolute errors were less than 0.6% for PDDs and OCRs

Abbreviations: AI, artificial intelligence; ML, machine learning; QA, quality assurance; DL, deep learning; PDD, percent depth dose; OPF, output factor; OCR, off-center ratio; ANN, artificial neural network; ARMA, autoregressive moving average; SVM, support vector machine; SVDD, support vector data description; kNN, k-nearest neighbor; EPID, electronic portal imaging device; MLC, multileaf collimator; MSE, square error.

## APPLICATION FOR PATIENT-SPECIFIC QUALITY ASSURANCE

For patient-specific QA, measurement- and calculation-based PSQA were recently recommended by AAPM TG218 and TG219 [13, 14]. In their reports, the tolerance limits of GPR, methodologies, and the effect of the MLC position accuracy are mentioned. For the application of AL and ML to patient-specific QA, many reports have been published on the prediction of mechanical errors in treatment machines and GPRs.

### Prediction and classification of mechanical errors in the treatment machine for patient-specific quality assurance

Table 2 shows a summary of the studies of the prediction or classification of mechanical errors of treatment machines for patient-specific QA. Input data included information obtained from the treatment plan and patient dose distribution. Output data included mechanical errors, including MLC, gantry, or MU, obtained from the treatment plan. Sakai *et al*. [26] detected MLC modeling parameters, including MLC transmission and dosimetric leaf gap (DLG), and MLC positional error for 38 beams from 19 clinical IMRT plans. They used five ML models, including decision tree, kNN, SVM, logistic regression and random forest to detect MLC transmission and DLG, as well as MLC positional errors from 837 radiomic features of the fluence difference map on EPID images. They observed that the sensitivity and specificity were obtained as 0.913 and 1.000 for MLC transmission error, 0.978 and 1.000 for DLG error, and 1.000 and 0.909 for MLC positional error using ML models. Using the GPR-based detection procedure, there was poor performance of sensitivity of 0.737 for MLC transmission and DLG errors and 0.882 for MLC positional error. They concluded that radiomics-based IMRT QA was a promising approach for detecting MLC parameter errors [29]. Kimura *et al*. [23] detected MLC positional error using CNN with dose difference maps of VMAT plans for prostate cancer. A total of 996 dose difference maps for input were created from 483 calculated dose distributions, and the performance of classification was evaluated. They observed that the classification accuracy, sensitivity and specificity were over 0.944, 0.889 and 0944, respectively. Furthermore, Kimura *et al.* reported error detection, including MLC positioning, gantry rotation, radiation output and phantom setup errors, modeled using CNN and VAE [25, 28]. They concluded that the ML approach has the potential to detect errors in patient-specific QA for VMAT.

**Table 2:** Summary of the studies on the prediction or classification of mechanical errors of the treatment machines for patient-specific QA

Reference	Year	Predicted or classified object	Learning model		Data set	Input	Main result
			Non-DL	DL			
Nyflot *et al.* [22]	2019	MLC position error	SVM, decision tree, and kNN	ANN	180 beams from 23 IMRT plans	588 gamma images	The highest classification accuracy of 64.3% was achieved with the SVM model
Kimura *et al.* [23]	2020	MLC position error	-	CNN	161 beams from 104 VMAT plans for prostate cancer	996 dose difference maps	The CNN model classified all errors with an accuracy of 0.944
Osman *et al.* [24]	2020	MLC position error	-	ANN	Over 400 log files containing 360 800 control points for IMRT plans	14 parameters	The prediction model achieved a maximum mean squared error of 0.0001 mm^2^ in predicting the leaf positions
Kimura *et al.* [25]	2021	Thirteen types of errors, including MLC position, gantry rotation, output, and phantom setup	-	CNN	161 beams from 104 VMAT plans for prostate cancer	3250 dose difference maps	The CNN model classified all errors with an accuracy of 0.92
Sakai *et al.* [26]	2021	MLC transmission, DLG, MLC position	Decision tree, SVM, kNN, logistic regression, and random forest	-	38 beams from 19 IMRT plans	837 radiomic features	Radiomic-based models showed a higher sensitivity of 1.000 and 0.090 for detecting MLC positional errors
Bedford *et al.* [27]	2022	MU, MLC aperture area, etc.	-	RNN	Six VMAT plans for prostate cancer	Four different metrics based on the difference between predicted and measured images	RNN reduced the rate of false negative results from 0.36 to 0.24
Kimura *et al.* [28]	2023	Nine types of errors, including MLC position, gantry rotation, output, and phantom setup	-	CNN VAE	161 beams from 104 VMAT plans for prostate cancer	720 dose difference maps	AUC of the VAE-based method, CNN and gamma analysis were 0.699, 0.921, and 0.669, respectively
Nakamura *et al.* [29]	2023	MLC transmission, DLG	-	CNN	33 clinical VMAT plans for prostate and head-and-neck cancer	360 subtracted dose distribution	The detection of MLC transmission and DLG error was feasible using Gaussian filters of 0.5

Abbreviations: QA, quality assurance; MLC, multileaf collimator; DLG, dosimetric leaf gap; MU, monitor unit; SVM, support vector machine; ANN, artificial neural network; kNN, k-nearest neighbor; CNN, convolutional neural network; RNN, recurrent neural network; VAE, variational autoencoder; IMRT, intensity-modulated radiation therapy; VMAT, volumetric-modulated arc therapy; AUC, area under the curve.

### Prediction of gamma passing rate for patient-specific quality assurance

Table 3 shows a summary of the studies of GPR prediction for patient-specific QA. Input data included information obtained from the treatment plan and patient dose distribution; plan complexity parameters, including modulation complexity score (MCS) or aperture area, were often used [77–79]. The GPRs obtained from the measured-based QA were predicted as output data. Various learning models, including Poisson regression, regression tree, random forest, gradient boost, ANN, CNN and GAN have been used, primarily using MATLAB (MathWorks, Inc., Natick, MA, USA) and Python as the development environments. Many reports mainly used plan complexity parameters or dose distribution as input data for predicting GPR. Ono *et al.* [33] predicted a GPR of 600 VMAT plans from 28 plan complexity parameters using regression tree, multi-regression and ANN . They observed that the prediction errors of GPR at 3%/ 3 mm were 0.6% ± 2.4%, 0.5% ± 2.4% and − 0.2% ± 2.1% for the regression tree, multi-regression and ANN, respectively. Wall *et al*. [35] predicted the GPR of 500 VMAT plans from 241 plan complexity parameters using eight types of learning models, including linear regression, Elastic Net, SVM, decision tree, random Forest, AdaBoost, gradient Boost and ANN. The 241 features can be categorized into 23 groups which mainly consist of MLC positions and MU. They found that the lowest mean absolute error (MAE) was 3.75% for GPR at 3%/3 mm using SVM. In their study, the ANN model performed slightly better than other models in terms of prediction error. In the recent report, GPR was predicted from plan complexity parameters for novel dual-layered MLC Linac [40]. As a multi-institutional study, Lambri *et al*. [37] predicted a GPR of 5522 VMAT plans to streamline the patient-specific QA process in three institutions. The GPR was predicted using XGBoost from 19 plan parameters, including 10 plan complexity, 6 dynamic and 3 static parameters. The prediction model was created based on the VMAT plans of one institution. They observed MAEs of three institutions were 2.33%, 2.54% and 3.91% for GPR at 3%/1 mm. They concluded that learning models trained within one institution may not be readily transferable to another, even when employing the same QA device.

**Table 3:** Summary of the studies on the prediction of GPR for patient-specific QA

Reference	Year	Learning model		Data set	Input	Prediction error
		Non-DL	DL			
Valdes *et al.* [30]	2016	Poisson regression	-	498 IMRT plans	78 plan complexity parameters	Errors within 3% for GPR at 3% / 3 mm
Valdes *et al.* [31]	2017	Poisson regression	-	637 IMRT plans	90 plan complexity parameters	Errors within 3.5% for GPR at 3% / 3 mm
Tomori *et al.* [32]	2018	-	CNN	60 IMRT plans	Volumes of PTV, OAR, MU, etc.	Errors within 1.1% for GPR at 3% / 3 mm
Ono *et al.* [33]	2019	Regression Tree, Multi Regression	ANN	600 VMAT plans	28 plan complexity parameters	Average error was −0.2% for GPR at 3% / 3 mm
Hirashima *et al.* [34]	2020	XGBoost	-	1255 VMAT plans	28 plan complexity parameters and 851 dosiomics features	MAE was 1.8% for GPR at 3% / 2 mm
Wall *et al.* [35]	2020	Linear regression, Elastic Net, SVM, Decision tree, Random Forest, AdaBoost, Gradient Boost	ANN	500 VMAT plans	241 plan complexity parameters	MAE was 3.75% for GPR at 3% / 3 mm
Tomori *et al.* [36]	2021	-	CNN	147 VMAT plans	Dose distribution of 96 dummy plans	MAE was 0.63% for GPR at 3% / 3 mm
Lambri *et al.* [37]	2023	XGBoost	-	5522 VMAT plans	19 plan parameters	MAEs of three institutions were 2.33%, 2,54%, and 3.91% for GPR at 3%/ 1 mm
Matsuura *et al.* [38]	2023	-	GAN	270 VMAT plans	Gamma distribution	RMSE was 1.0% for GPR at 3% / 2 mm
Tozuka *et al.* [39]	2023	-	ANN	96 VMAT plans	MLC position map and dose distribution	MAE was 1.25% for GPR at 3% / 3 mm
Zhu *et al.* [40]	2023	Gradient boosting decision tree, random forest, Poisson regression	-	213 IMRT plans	33 plan complexity parameters	The average error was 0.78% for GPR at 3%/ 2 mm

Abbreviations: GPR, gamma passing rate; QA, quality assurance; DL, deep learning; CNN, XGBoost; eXtreme Gradient Boosting, convolutional neural network; ANN, artificial neural network; SVM, support vector machine; generative adversarial network; MLC, multileaf collimator; IMRT, intensity-modulated radiation therapy; VMAT, volumetric-modulated arc therapy; PTV, planning target volume; OAR, organ at risk; MU, monitor unit; MAE, mean absolute error; RMSE, root mean square error.

In terms of input data for dose distribution, Tozuka *et al.* [39] predicted the GPR from MLC position maps and dose distribution using an ANN. They compared three models, namely, dose distribution in the patient geometry combined with MLC positions (model 1), dose distribution in the patient geometry (model 2), and dose distribution on a helical diode array (model 3). They found that Model 1 had the lowest MAE of 1.25% for GPR at 3%/ 3 mm. Matsuura *et al.* [38] predicted GPR using a synthesized gamma distribution for 270 VMAT plans using a deep convolutional generative adversarial network (GAN). The gamma distributions were acquired from the calculated fluence map and the measured map on the EPID. They found that the root-mean-squared errors of the GPRs were 1.0%, 2.1%, 3.5% and 3.6% at 3%/ 2 mm, 2%/ 1 mm, 1%/ 1 mm and 1%/ 0.5 mm, respectively. GPR prediction is expected to contribute to the efficiency and reduce the workload of patient-specific QA. Chen *et al.* [80] have also reported on classifying GPR using non-DL methods, a local interpretable model-agnostic explanation framework, and a novel adaption of team-based Shapley values framework. Regarding other aspects of the prediction of GPR, Anetai *et al*. [81] reported that one of the non-DL methods of lie derivative image analysis could extract gradient-induced problematic regions of GPR failure points. Their study indicated that ML can expand the possibility of the traditional method, that is the non-DL method, to cover up weakness.

## FUTURE DIRECTIONS AND CHALLENGES

While many types of studies have reported the application of AI for machine- and patient-specific QA, it is expected that more reports on the expansion to practical clinical applications, including prospective and multi-institutional studies, will be reported in the future.

To enable the clinical application of QA methods employing AI, AI models must exhibit versatility, constancy, and accessibility. For versatility and constancy, Yang *et al.* [82] reported a clinical implementation study of a multi-institution scenario. In their study, they confirmed the versatility of the QA model in multiple scenarios for different delivery equipment, QA devices, and TPS in different institutions. In addition, the predicted and measured results were evaluated using an end-to-end test to check the consistency of clinical implementation. For accessibility, Wall *et al.* [83] reported prospective clinical validation of virtual patient-specific QA for VMAT using GPR prediction with XGBoost . They prospectively predicted the GPR for 445 VMAT plans over 3 months at one institution. They observed that predicted GPR had a mean absolute error of 1.08% ± 0.77%. Using a 1% prediction threshold, they estimated that the QA workload would yield a 69.2% reduction and save 32.5 hours per month. The introduction of a prediction-based QA method, rather than a conventional measurement-based QA, is expected to enhance the efficiency of the QA workload. In addition, considering the current status of radiotherapy in recent years, online adaptive radiation therapy has been increasingly adopted [84]. As for its QA approach, performing measurement-based QA is difficult, necessitating the implementation of calculation-based QA. However, calculation-based QA cannot evaluate equipment performance or actual dose distribution obtained through measurement verification, and may not necessarily reflect the results of measurement-based QA. We believe that QA prediction methods can predict the results of measurement-based QA, including the generalized performance results obtained through measurement verification, which cannot be reflected in calculation-based QA. In a multi-institutional study, Kelendralis *et al*. [85] reported a Bayesian network (BN)-based initial plan review assistive tool as automatic patient-specific QA in a multi-institution. They collected data from 17 666 patients from three institutes in Europe (8753 patients) and two institutions in the United States (8913 patients). They trained a BN model to detect three error categories, specifically, patient setup, treatment planning and prescriptions. They found that the highest performance was observed with an AUC of 0.991 for MU. They concluded that the BN model has performed well even in different international institutions. To predict the GPR for VMAT plans, prospective clinical validation and multi-institutional studies would allow the reliability and validity of the technique to be assessed and contribute significantly to its future dissemination.

New methods, such as beam data prediction, MLC position prediction for irradiation plans, and GPR prediction, have become feasible through the use of AI. Further challenges in innovative methods based on AI applications have been reported. Wall *et al.* [41] reported a new optimization approach based on predicted GPR for 13 prostate VMAT plans. They modified the mechanical treatment features associated with predicted GPRs and achieved optimal VMAT plans. They observed that the predicted GPRs at 3%/ 3 mm increased by an average of 1.14 ± 1.25% using this approach. Ono *et al*. [42] developed a plan complexity mitigation algorithm based on the predicted GPR for 50 VMAT plans. They modified the MLC position to increase the predicted GPRs to 5% / 1 and 3%/ 2 mm, using a limited nonlinear algorithm. They found that plan complexity parameters were decreased by 0.8 ± 1.7 (×10^−2^) and 42.7 ± 57.9 mm^2^ for MCS applied for VMAT and average aperture area, respectively. For measurement dose with ArcCHECK cylindrical diode array (SunNuclear, Melbourne, FL, USA), they observed that mitigated VMAT plans improved GPRs with 1.8 ± 2.9% at 5%/1 mm and 1.3 ± 1.8% at 3%/2 mm compared with that of original VMAT plans. Yang *et al.* [43] developed a new patient-specific QA approach using an uncertainty-guided man–machine integrated approach. They employed a dual-task DL model that incorporated uncertainty awareness, along with an interwoven training approach and Monte Carlo dropout for Bayesian inference. This combination facilitates the simultaneous generation of the predicted GPR and the corresponding total prediction uncertainty, thereby guiding patient-specific QA. They used 1541 pairs of field fluences and GPRs for modeling and observed a clinical accuracy of 100.0% with only 61.7% workload. Their new method addresses the issue of uncertainty, which is not considered in currently reported AI-based GPR prediction models. This was developed as one of the motivations for conducting this research. Therefore, the use of state-of-the-art technologies is expected to lead to further technological development.

This article reviews the current status of AI applications in machine- and patient-specific QA and discusses future directions. The input data included the beam data, dose distribution and plan parameters. Various types of features are used for prediction and classification, and feature extraction and selection methods should be considered depending on the situation. Data training, validation, and testing are also necessary for robust modeling. Particularly, in machine- and patient-specific QA, the number of datasets is often small, and it is important to contemplate suitable validation methods. Supervised learning techniques, such as XGBoost, and DL models, such as ANN and CNN, are widely used as learning models. In addition, more advanced models, such as LightGBM and GAN, have been used. At the time of the survey phase (December 2023), there were fewer reports on AI applications for machine-specific QA than for patient-specific QA. Patient-specific QA involves several types of data, and it would be interesting to use AI to improve the efficiency of QA methods. It is expected that more advanced learning models will be developed and utilized in the future for both machine- and patient-specific QA, including new plan optimization approaches as reported by Wall *et al*. [41] and Ono *et al.* [42] or a new patient-specific QA approach as reported by Yang *et al.* [43] Furthermore, we believe that efficiency and generality of radiotherapy workflow all will be achieved by AI-based systems. In facilities with a large number of radiotherapy patients, efficiency leads to medical safety. In small-scale institutions with few radiotherapy patients, the generalization of tasks through AI enables the confident provision of medical care. With the development of AI, the field of radiation therapy has also undergone various technological developments. While innovative AI technology is expected to continue to develop, its use and application must be carefully understood when seeking new challenges for machine- and patient-specific QA in radiation therapy.
